# Complete Genome Sequence of *Bacillus megaterium* Bacteriophage Eldridge

**DOI:** 10.1128/genomeA.01728-15

**Published:** 2016-04-21

**Authors:** Alexandra M. Reveille, Kimberly A. Eldridge, Louise M. Temple

**Affiliations:** Department of Integrated Science and Technology, James Madison University, Harrisonburg, Virginia, USA

## Abstract

In this study the complete genome sequence of the unique bacteriophage Eldridge, isolated from soil using *Bacillus megaterium* as the host organism, was determined. Eldridge is a myovirus with a genome consisting of 242 genes and is unique when compared to phage sequences in GenBank.

## GENOME ANNOUNCEMENT

Bacteriophage Eldridge is a member of the *Myoviridae* family, isolated on *Bacillus megaterium* as the host organism. *B. megaterium* is a Gram-positive, rod-shaped, endospore-forming bacterium. *B. megaterium* is a nonpathogenic host used for the biotechnological production of substances, including vitamin B_12_, amylases, and penicillin amylase (1). Eldridge was discovered in 2014 in soil from the James Madison University campus (GPS coordinates 38°25′58.4″N 78°51′43.5″W), having been isolated after soil enrichment with *B. megaterium*.

Phage genomic DNA was submitted to the North Carolina State Genomic Sciences Laboratory (Raleigh, NC, USA) for Illumina library construction and sequencing, which was performed using a 150-bp single-end sequencing flow cell with a MiSeq reagent kit version 3 (Illumina, USA). Using 454 Newbler version 2.9 (2), the raw data were assembled into one contig. Average coverage was found to be 200-fold using 49,597 reads. Gene prediction was completed using GeneMarkS (3) and Glimmer (4). Eldridge was autoannotated and refined using DNAmaster (http://cobamide2.bio.pitt.edu/computer.htm). Functions were predicted after analysis with protein BLAST (5) and HHPRED (http://toolkit.tuebingen.mpg.de/hhpred/help_ov). tRNAgenes were annotated using Aragorn (http://130.235.46.10/ARAGORN).

The Eldridge genome consisted of 162,418 bases, with a GC content of 40%. Like many other *Bacillus* phages, the genome contained a direct terminal repeat of 3,199 bp (6), determined by the occurrence of double coverage with well-defined margins in the assembled contig. Fifty-seven genes encoding functional proteins were putatively identified. Of these, 14 structural proteins were identified, including tail sheath proteins, capsid proteins, and baseplate proteins. Several genes were found to code for lytic proteins, including holin, autolysins, and tail lysins. Additionally, 10 proteins utilized in DNA metabolism, 8 proteins utilized in DNA replication, and 9 proteins predicted to affect gene regulation were identified. Unlike its closest relatives and other reported *Bacillus* phages, the DNA polymerase did not show frame shifts or the presence of introns. Eldridge encodes for a sporulation sigma factor SigF, suggesting that the phage may impact host expression of sporulation genes (7). The genome was found to contain 3 tRNA genes.

Eldridge was classified as a member of the *Myoviridae* family because it has a contractile tail. Transmission electron microscopy revealed a symmetrical head with an approximate diameter of 60 nm and an extended tail length of approximately 180 nm. Using BLASTn analysis (8), the closest relative found was *B. megaterium* phage Moonbeam (9), which related only to 42% distributed along the genome.

### Nucleotide sequence accession number.

The complete genome of the bacteriophage Eldridge is deposited in GenBank under the accession number KU253712.
